# Integrated multi-omics profiling to dissect the development of second primary lung cancer in laryngeal cancer: An observational study

**DOI:** 10.1097/MD.0000000000047679

**Published:** 2026-03-06

**Authors:** Fengfeng Xu, Tengfei Huang, Qianhui Xu, Meiqing Zhang, Shiyan Chen

**Affiliations:** a Department of Cardiothoracic Surgery, 900 Hospital of the Joint Logistic Team, Fuzhou, People’s Republic of China; b Department of Otolaryngology, Fujian Medical University Union Hospital, Fuzhou, People’s Republic of China.

**Keywords:** immune cell infiltration, laryngeal cancer, metabolic analysis, second primary lung cancer, single-cell RNA sequencing

## Abstract

Laryngeal cancer, a prevalent malignant tumor, frequently leads to mortality through the development of second primary lung cancer (SPLC). To investigate the potential mechanisms underlying SPLC development in laryngeal cancer patients, we conducted an integrated multi-omics analysis. We obtained laryngeal cancer (GSE51985) and lung cancer (GSE102287) datasets from the gene expression omnibus and identified differentially expressed genes using the “limma” package. Our analysis revealed 9 shared genes, with UBE2C, POLQ, RAD51, and HOXB7 being up-regulated and EDNRB, GPD1L, F10, SORBS2, and CXCL12 down-regulated in both cancers. Functional enrichment analysis indicated these genes were primarily involved in pathways in cancer and the cell cycle. We further constructed transcription factor (TF)–miRNA–gene interaction networks, identifying 120 TFs and 246 miRNAs coordinating these shared genes. Metabolic analysis linked CXCL12 to inositol phosphate metabolism, and single-cell RNA sequencing from datasets GSE150321 and GSE127471 demonstrated that intermediate monocytes in lung cancer were highly active in this metabolic pathway. Additionally, immune cell infiltration analysis using CIBERSORT revealed a higher proportion of macrophages in both cancer types compared to non-tumor tissues. In conclusion, our study suggests that shared genetic alterations, regulated by specific TFs and miRNAs, alongside an altered immune microenvironment and CXCL12-mediated inositol phosphate metabolism likely driven by intermediate monocytes, contribute to the development of SPLC following laryngeal cancer.

## 1. Introduction

Laryngeal cancer is a prevalent malignant tumor in the head and neck region, comprising approximately 20% of all head and neck tumors and about 1.5% of adult cancers.^[1,2]^ Recent statistics indicate an increase in both incidence and mortality rates. In 2023, there were 12,380 newly diagnosed cases and 3820 deaths related to laryngeal cancer in the United States.^[3]^ Among these cases, squamous cell carcinoma accounts for 85% to 95% of the pathological types.^[4]^ The 5-year survival rate for laryngeal cancer remains below 50%, indicating a poor prognosis.^[5]^ However, some scholars have proposed that early detection of laryngeal cancer can significantly improve the 5-year survival rate to 90%.^[6]^ Despite comprehensive treatments, patients with advanced disease still face a higher risk of recurrence, contributing to the low survival rate.^[7]^ Epidemiological studies have identified the occurrence of second primary cancer as the primary cause of death in laryngeal cancer cases, accounting for approximately 21%.^[8,9]^ Notably, more than 50% of these cases involve second primary lung cancer, as highlighted in over 9 studies.^[10,11]^

Adams et al conducted a study on 209 patients with laryngeal cancer and found that within a 5-year period, 8% of patients developed second primary lung cancer, which is equivalent to an annual incidence rate of 1.6%.^[12]^ Another study reported that regardless of the stage of cancer, the incidence of secondary primary lung cancer within 5 years can be as high as 30%.^[13]^ In a joint study involving 13 national cancer centers, it was observed that squamous cell carcinoma of the head and neck resulted in a 13% incidence of second primary lung cancer over a 20-year period. Multiple studies have consistently shown that patients with second primary lung cancer following laryngeal cancer have a significantly lower survival rate compared to those with first primary lung cancer, with a median survival time of 7 months.^[12,14,15]^ Laryngeal cancer patients are more likely to have risk factors for second primary lung cancer, such as smoking, alcohol consumption, and HPV virus infection.^[12,16–18]^ The location of the laryngeal cancer may also contribute to the increased incidence of second primary lung cancer. Clinical studies using the SEER database have demonstrated that radiotherapy after a diagnosis of laryngeal cancer can further increase the incidence of second primary lung cancer by 18% compared to patients who did not undergo radiotherapy.^[12]^

Previous studies have extensively investigated the clinical risk factors associated with the development of second primary lung cancer in laryngeal cancer. Furthermore, the identification of genetic markers for both laryngeal and lung cancer has enabled effective prediction of the diagnosis and prognosis of laryngeal cancer.^[19,20]^ Nowadays, many bioinformatics strategies have been employed to explore the progression of tumors.^[21,22]^ Single-cell RNA sequencing technology has been widely utilized to classify tumor cell types and determine tumor consistency, cellular properties, novel markers, as well as molecular and functional strategies.^[23,24]^ In this study, we aim to explore the shared gene markers of laryngeal and lung cancer, investigate the tumor microenvironment, and examine the role of intratumor microorganisms in the occurrence and progression of the disease. By deepening our understanding of this disease, we can provide improved treatment options, reduce morbidity, and enhance survival rates.

## 2. Materials and methods

### 2.1. Data download and processing

Datasets for non-small cell lung cancer were downloaded from the gene expression omnibus (GEO) database (Home-GEO-NCBI [nih.gov]). The selection of a specific GEO dataset was mainly based on the availability and relevance of the data. We chose the dataset that contained samples of laryngeal cancer and lung cancer to ensure that we could analyze the shared genes between the 2 types of cancer. Specifically, data from GSE102287 includes 245 non-small cell lung cancer samples and normal controls were used. For laryngeal cancer analysis, the GSE51985 datasets were utilized, which included tumor tissues and paired adjacent tissues from 10 patients with primary laryngeal cancer. The “limma” package in R-4.3.0 software was employed to identify differentially expressed genes (DEGs) based on criteria of *P* < .05 and |log 2 (FoldChange)| > 1. The GSE157010 and GSE59102 datasets were used for the validation. Due to the limited sample size of the laryngeal cancer dataset (GSE51985), we employed a combination of a *P*-value threshold (*P* < .05) and a stringent foldchange criterion (|log_2_ FC| > 1) to identify DEGs without applying multiple testing correction, aiming to reduce false positives while maintaining sensitivity. The volcano plot was generated using the “ggplot2” package. DEGs were intersected using Venny 21.0 (Venny 2.1.0 [csic.es]) to obtain common genes. Correlation analysis and heat mapping of the common genes were performed using the online bioinformatics analysis and visualization cloud platform called Network (bioinformatics.com.cn) to identify key genes.

### 2.2. Functional enrichment analysis and construction of protein interaction networks

To identify differential genes, we found 9 genes that intersected. For gene set functional enrichment analysis, we utilized the KEGG rest API (KEGG API manual) to obtain the most recent gene annotations of the KEGG pathway. We used these annotations as the background set, mapped the genes onto this background set, and performed enrichment analysis using the R software package clusterProfiler (version 3.14.3) to obtain the results of gene set enrichment. We considered a minimum gene set of 5 and a maximum gene set of 5000, with a *P* value of <.05 and a false discovery rate of <0.25 being considered statistically significant. To generate protein–protein interaction networks (PPIs) for the different generated genes, we utilized the STRING website (STRING: functional protein association networks [string-db.org]). The data was downloaded from the database in TSV format and visualized using Cytoscape_v3.9.0 software. Additionally, we analyzed gene mutations of key genes using the cBioPortal (cBioPortal for Cancer Genomics) for Cancer Genomics database. We examined the recurrence of laryngeal cancer patients and the survival of lung cancer patients in both the mutated and non-mutated groups.

### 2.3. Constructing transcription factor-miRNA-gene interactions

Transcription factor and miRNA analysis of shared genes was performed using the NetworkAnalst database (NetworkAnalyst) , the data were downloaded, and visualized with Cytoscape_v3.9.0 software.

### 2.4. Shared gene diagnostic analysis

SPSS version 23.0 was utilized to analyze the receiver operating characteristic curve (ROC analysis) of the clinical data from the GSE102287 and GSE51985 datasets. The area under the curve (AUC) was plotted to assess the diagnostic sensitivity and specificity of genes in both lung cancer and laryngeal cancer. Next, we performed pathological analysis using the ProteinAtlas database (The Human Protein Atlas).

### 2.5. Analysis of lipopolysaccharide-related genes in bacteria

The genes related to bacterial lipopolysaccharide were obtained from the CTD Database (The Comparative Toxicogenomics Database|CTD [ctdbase.org]) and the shared genes were identified using Venny 21.0 (Venny 2.1.0 [csic.es]). The main metabolic pathways of CXCL12 were analyzed using the MetaboAnalyst website (MetaboAnalyst) .

### 2.6. Single-cell population characterization

To further investigate the relationship between tumor cells and the tumor microenvironment, we utilized 10× single-cell RNA sequencing (scRNA-seq) data from lung cancer GSE127471 (GSM3635372) and single-cell data from Laryngeal Cancer GSE150321 (GSM 45466858). We performed data collation, normalization, and principal component analysis using the “Seurat,” “patchwork,” and “dplyr” packages. Initially, we filtered the scRNA-seq data to retain high-quality cells based on the following criteria: Elimination of cells with more than 10% mitochondrial genes; Selection of cells expressing <1000 genes. Cell cluster analysis was conducted using the T-distribution stochastic neighborhood embedding algorithm. Subsequently, we employed the “SingleR” package for cell annotation. After visualizing the cell population, we utilized the “scMetabolism” package to analyze the metabolic pathway and compare the expression of the cell population in Inositol phosphate metabolism.

### 2.7. Immune cell infiltration analysis

The infiltration status of 22 different immune cells was assessed by the “CIBERSORT” package to explore the major immune invasions in lung cancer and Laryngeal cancer.

### 2.8. Ethical approval

This study is a bioinformatics analysis based on publicly available data from the GEO database. Since it did not involve direct interaction with human subjects or the use of private clinical samples, ethical approval from an institutional review board and informed consent were not required for this study.

## 3. Results

### 3.1. Identify shared genes for laryngeal and lung cancers

**Figure 1.**
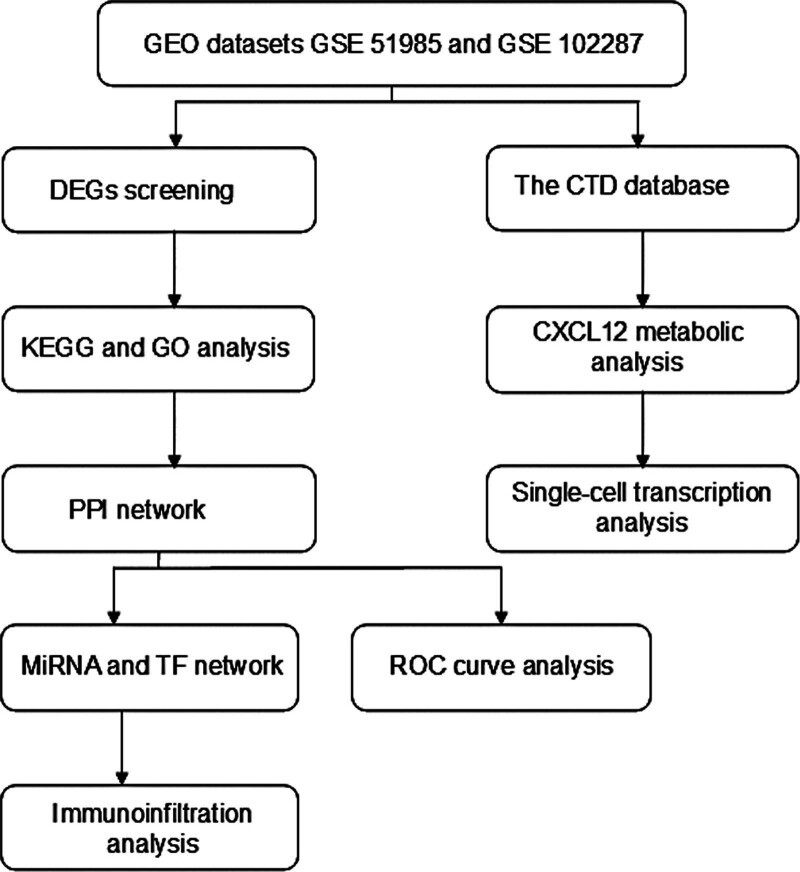
The workflow of this study.

**Figure 2.**
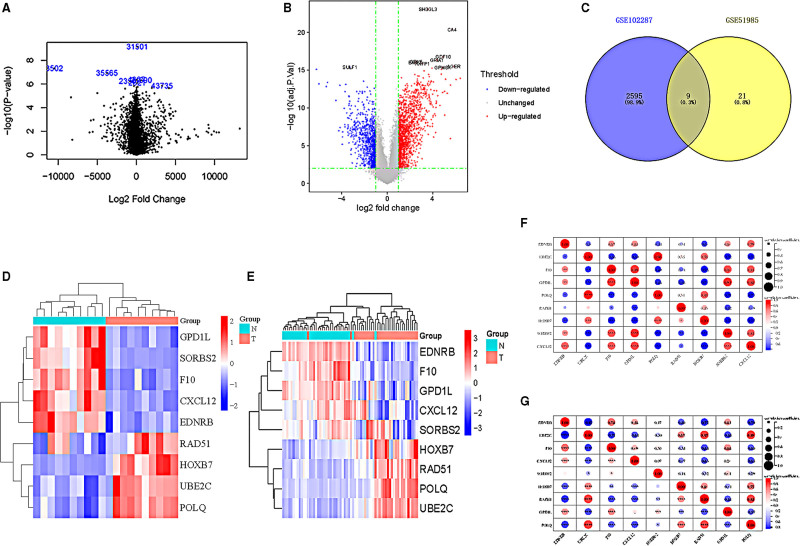
Identification of shared genes between laryngeal cancer and lung cancer. (A–C) Volcano plots of differentially expressed genes in laryngeal cancer (A) and lung cancer (B), and a Venn diagram (C). (D–E) Heatmaps of shared genes in laryngeal cancer (D) and lung cancer (E). (F–G) Identification of the correlation of shared genes in laryngeal cancer (F) and lung cancer (G).

The workflow of the study was shown in Figure 1. First, using |logFC|>1 and *P*-value < .05 as thresholds, we identified a total of 17 up-regulated DEGs and 13 down-regulated DEGs from the GSE51985 datasets (Fig. 2A). Similarly, we found 2604 DEGs in the GSE102287 dataset, with 1603 up-regulated DEGs and 1001 down-regulated DEGs (Fig. 2B). The intersection of these datasets revealed 9 shared genes, including 5 up-regulated genes (EDNRB, GPD1L, F10, SORBS2, and CXCL12) and 4 down-regulated genes (UBE2C, POLQ, RAD51, and HOXB7) (Fig. 2C). Heat maps of the shared genes demonstrated up-expression levels of UBE2C, POLQ, RAD51, and HOXB7 in laryngeal and lung cancers, while EDNRB, GPD1L, F10, SORBS2, and CXCL12 were down-expressed (Fig. 2D and E). Furthermore, the correlation analysis of the shared genes indicated a strong positive correlation (correlation coefficients > 0.9) between POLQ and UBE2C in both lung and laryngeal cancers (Fig. 2F and G).

### 3.2. Functional enrichment analysis and PPI network for DEGs

**Figure 3.**
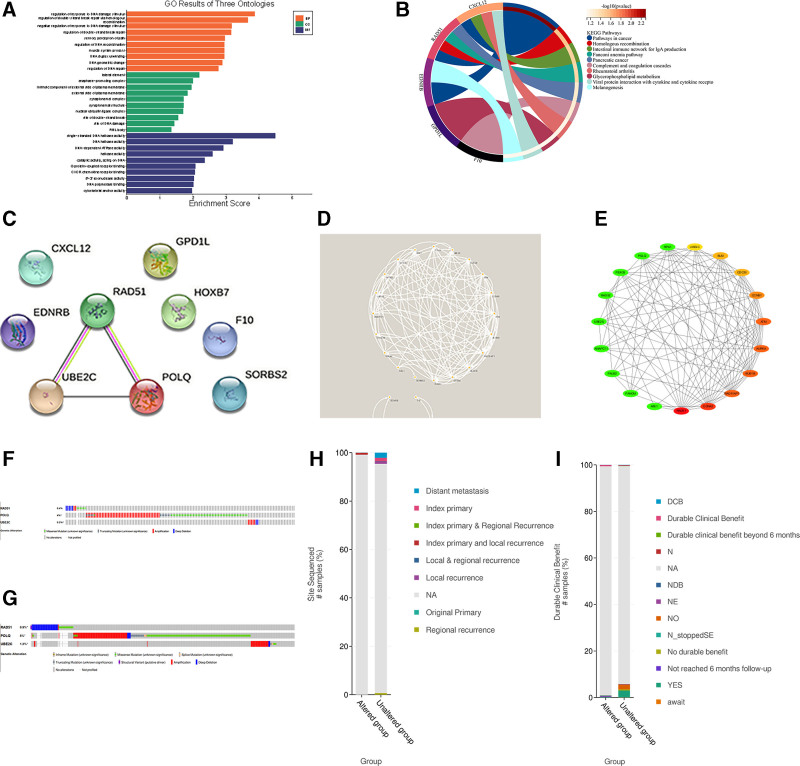
Functional enrichment analysis of DEGs and construction of PPI network. (A, B) GO (A) and KEGG (B) showing functional enrichment analysis of shared genes; (C–E) PPI network diagram of shared genes (C), protein interactions are extended to 3 times and visualized using Cytoscape, identification of critical subnets using MCODE plug-in (D, E). (F–I) RAD51, UBE2C, and POLQ mutations in laryngeal cancer (F) and lung cancer (G), and comparison of clinical data between mutant and non-mutant groups in laryngeal cancer (H) and lung cancer (I).

GO enrichment analysis is commonly used to investigate interactions between genes and pathways, while KEGG enrichment analysis can demonstrate the relationship between genes and functional patterns. The results of the GO analysis revealed that the notable pathway in biological processes was regulation of response to DNA damage stimulus, and lateral element in cellular component. In terms of molecular function, the statistically significant GO pathway was single-stranded DNA helicase activity. The first pathway identified in the KEGG enrichment analysis was Pathways in cancer, followed by Homologous recombination and Intestinal immune network for IgA production (Fig. 3A and B). These findings suggest a strong association between these DEGs and tumorigenesis as well as the cell cycle, which could provide a deeper understanding of laryngeal cancer and second primary lung cancer. To explore protein interactions, a comprehensive analysis of DEGs between laryngeal and lung cancers was performed using STRING. Figure 3C illustrates the interaction between DEGs that are common in both laryngeal and lung cancers, with RAD51, POLQ, and UBE2C being closely linked (Fig. 3C). Protein interactions were expanded up to 3 times and visualized using Cytoscape, and key subnets were identified using the MCODE plug-in. RAD51 emerged as the most prominent key gene, while UBE2C and POLQ were identified as minor key genes (Fig. 3D and E). In the cBioPortal database, mutation analysis of RAD51, UBE2C, and POLQ was conducted, revealing that RAD51 primarily exhibited deep deletion in both laryngeal and lung cancers, while POLQ showed a missense mutation of unknown significance followed by amplification, and UBE2C exhibited amplification (Fig. 3F and G). Further in-depth analysis demonstrated that the mutated group in laryngeal cancer was more likely to develop primary cancer and local recurrence compared to the non-mutated group. In lung cancer, it was observed that the mutated group experienced death within <6 months of follow-up (Fig. 3H and I). These findings also suggest that laryngeal cancer is followed by the development of a second primary lung cancer, ultimately leading to death.

### 3.3. Construct a PPI network of transcription factors and miRNAs

**Figure 4.**
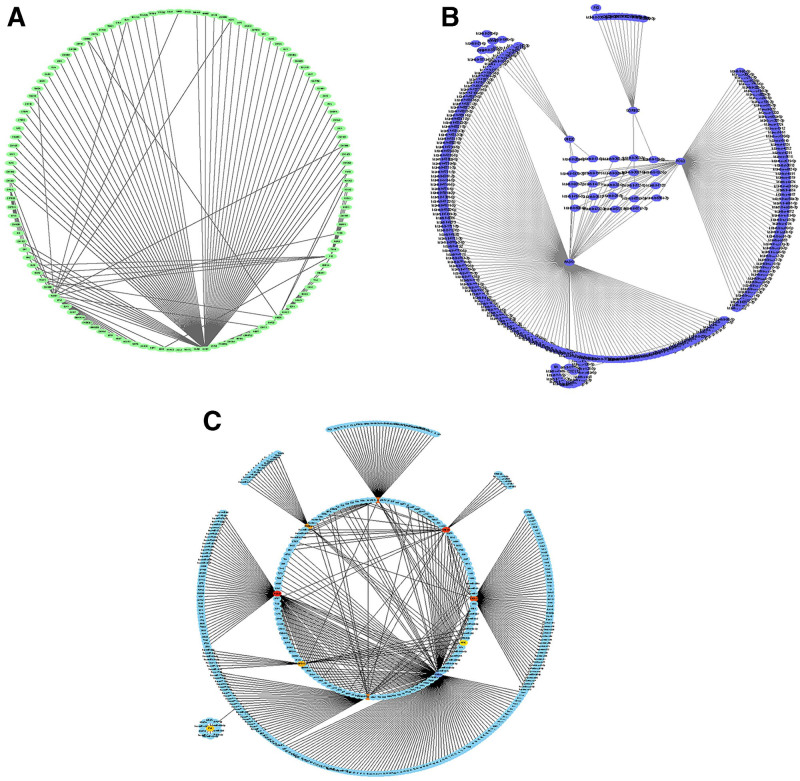
PPI networks of transcription factors and miRNAs. Transcription factor PPI network (A), miRNA PPI network (B), and TFs-miRNA PPI network diagram (C).

There are 2 types of gene expression regulators: transcription factors (TFs) and miRNAs. TFs regulate transcription by binding to promoter regions, while miRNAs regulate posttranscriptional gene expression. The analysis of the interaction between TFs and miRNAs revealed that 120 TFs and 246 miRNAs coordinated the expression of these common DEGs, indicating a close collaboration between them. The top 10 TFs, including TFDP1, EZH2, MAZ, ZNF324, HDAC2, HDAC6, GLIS2, RCOR2, KDM1A, and ZNF580, were sorted based on the *P* value. Similarly, the top 10 miRNAs were hsa-mir-193b-3p, hsa-mir-17-5p, hsa-mir-20a-5p, hsa-mir-32-5p, HSA-mir-92a-3p, hsa-mir-192-5p, hsa-mir-196a-5p, hsa-mir-129-5p, hsa-mir-221-3p, and hsa-mir-142-3p. The findings were visualized using Cytoscape (Fig. 4A–C). These results suggest a close relationship between common DEGs, transcription factors, and miRNAs.

### 3.4. ROC curve analysis of shared genes

**Figure 5.**
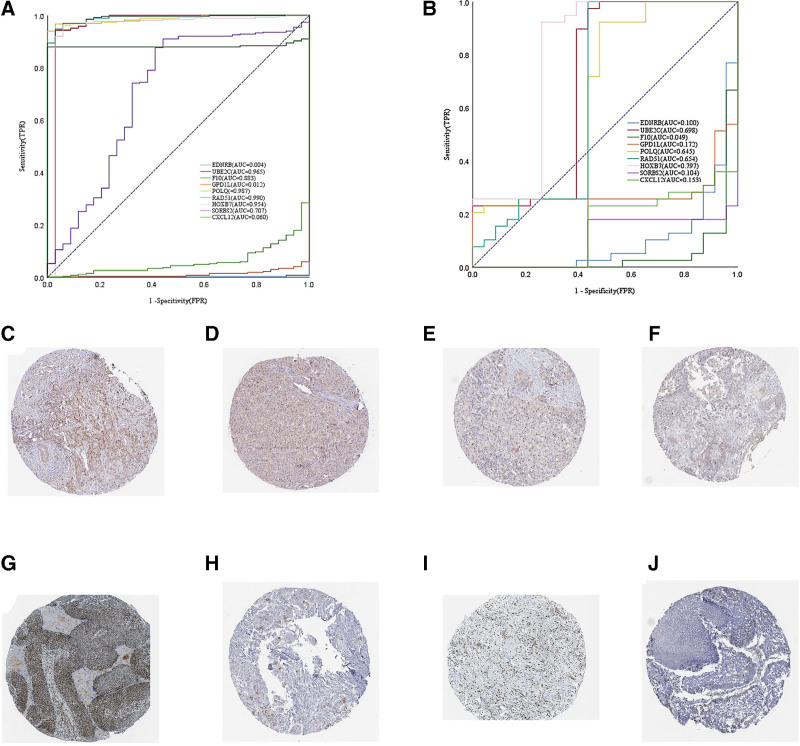
ROC curve analysis of shared genes. The ROC curve of 9 shared genes in laryngeal (A) and lung cancers (B). RAD51 and UBE2C is strongly stained in pathological tissues of laryngeal cancer (C–G), and lowly stained in pathological tissues of laryngeal cancer (D–H). RAD51 and UBE2C is strongly stained in pathological tissues of lung cancer (E–I), and lowly stained in pathological tissues of lung cancer (F–J).

ROC analysis was performed on the shared genes in the GSE157010 and GSE59102 datasets. The analysis revealed that the AUC for UBE2C, F10, POLQ, RAD51, HOXB7 and SORBS2 in lung cancer were above 0.7, while the AUC values for other genes were under 0.7. In laryngeal cancer, the AUC values for UBE2C, POLQ, RAD51, and HOXB7 were above 0.65. These results suggest that these genes could potentially serve as new diagnostic markers for laryngeal and lung cancer (Fig. 5A and B). RAD51 and UBE2C is strongly stained in pathological tissues of laryngeal cancer (Fig. 5C and G), and lowly stained in pathological tissues of laryngeal cancer (Fig. 5D and H). RAD51 and UBE2C is strongly stained in pathological tissues of lung cancer (Fig. 5E and I), and lowly stained in pathological tissues of lung cancer (Fig. 5F and J).

### 3.5. CXCL12 metabolic analysis

**Figure 6.**
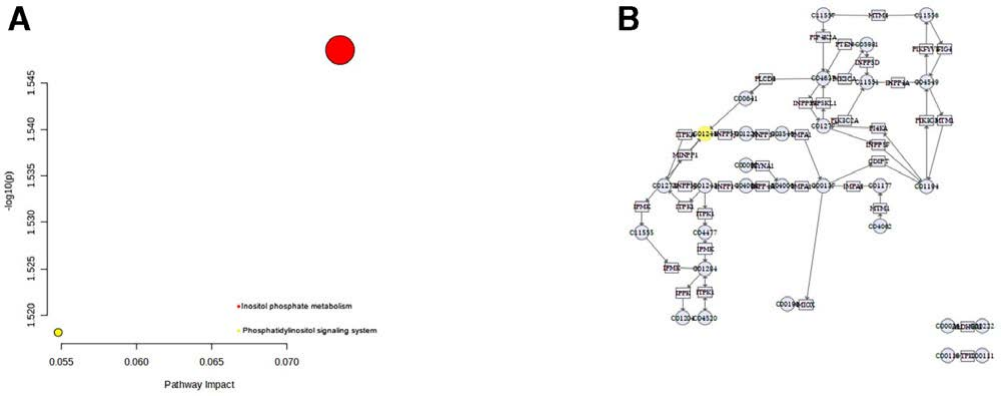
Metabolic analysis of CXCL12. The MetaboAnalyst website indicated that inositol phosphate metabolism and the phosphatidylinositol signaling system are the metabolic pathways associated with CXCL12 (A), and the pathway of inositol phosphate metabolism (B).

The bacterial lipopolysaccharide (LPS)-associated gene was downloaded from the CTD database and intersected with the shared genes. This analysis revealed that CXCL12 is the main gene associated with laryngeal cancer, lung cancer, and tumor microbiota. Additionally, the metabolic analysis of CXCL12 conducted using the MetaboAnalyst website indicated that inositol phosphate metabolism and the Phosphatidylinositol signaling system are the metabolic pathways associated with CXCL12. Notably, inositol phosphate metabolism was identified as the primary metabolic pathway (Fig. 6A and B).

### 3.6. Single-cell RNA sequencing analysis

**Figure 7.**
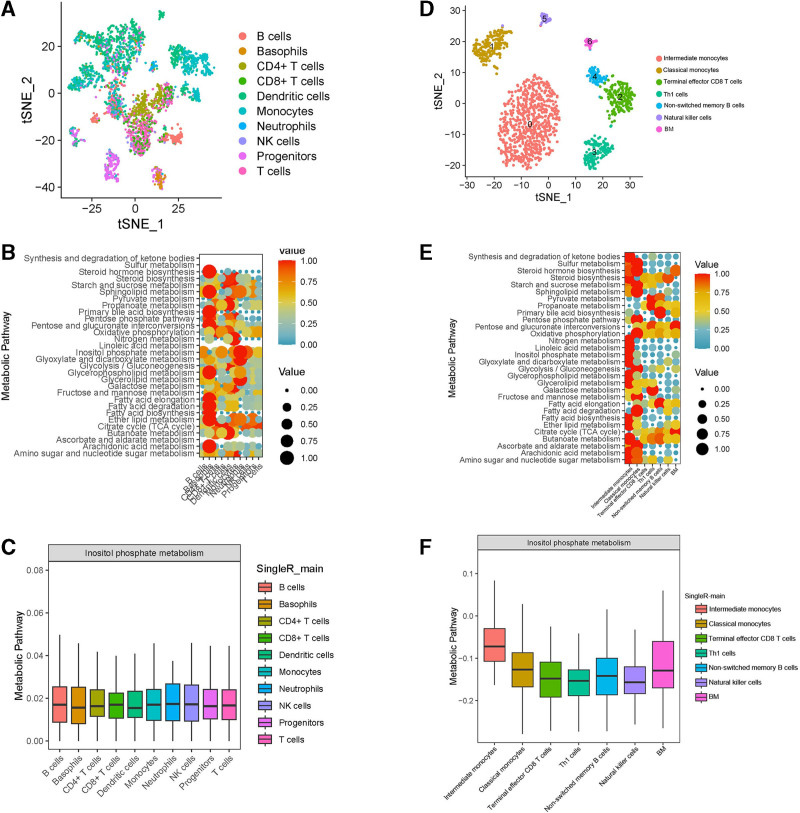
Single-cell sequencing analysis of the GEO datasets. GSE150321 (A) and GSE127471 (D) recognize cell populations; metabolic heatmaps in laryngeal cancer (B) and lung cancer (E); box diagram of cell populations in metabolic pathways in laryngeal cancer (C) and lung cancer (F).

After processing and screening scRNA-seq data, we performed principal component analysis on the gene expression profile of laryngeal cancer samples to reduce dimensionality and identified 13 cell clusters. Using the “SingleR” package, we annotated the cell identity of each cluster, which included B cells, Basophils, CD4+ T cells, CD8+ T cells, dendritic cells, monocytes, neutrophils, NK cells, progenitors, and T cells (Fig. 7A). Similarly, in lung cancer cell clustering, we found Intermediate monocytes, classical monocytes, terminal effector CD8 T cells, Th1 cells, non-switched memory B cells, natural killer cells, and BM (Fig. 7D). Metabolic heat map analysis of laryngeal cancer and lung cancer cell populations revealed that intermediate monocytes may be involved in most metabolic pathways in lung cancer, while Basophils may be involved in most metabolic pathways in laryngeal cancer (Fig. 7B and E). Further analysis showed that Intermediate monocytes in lung cancer were more active and expressed more components than other cells during inositol phosphate metabolism. However, there was no significant difference in CXCL12-mediated inositol phosphate metabolism in laryngeal cancer (Fig. 7C and F). Abnormalities in the CXCL12-related inositol metabolism pathway may create a tumor-promoting microenvironment to the development of secondary primary lung cancer.

### 3.7. Immunoinfiltration analysis

**Figure 8.**
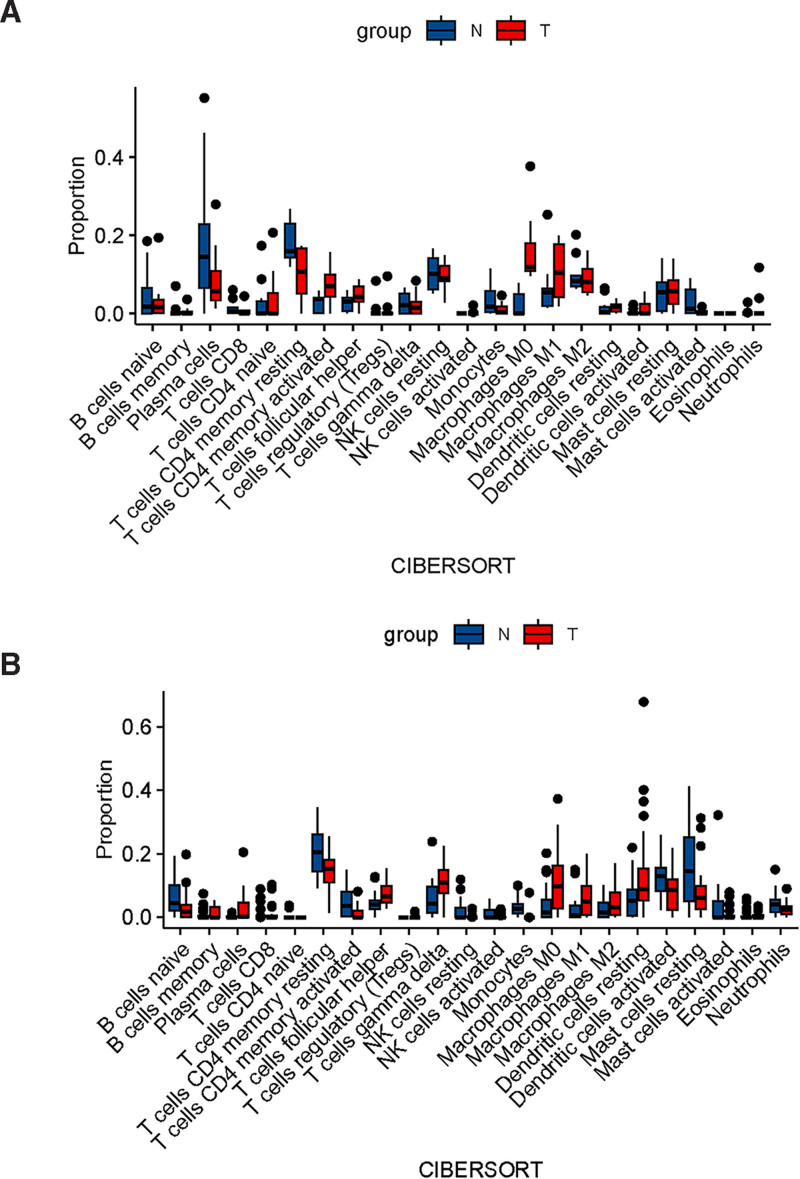
Analysis of immune cell infiltration. Box diagrams of immune cells in laryngeal cancer (A) and lung cancer (B).

The single-cell analysis revealed a close relationship between immune cells and tumors. We conducted immune invasion analysis separately for laryngeal cancer and lung cancer. The findings showed that in lung cancer, B cells naïve, T cells CD4 memory resting, T cells CD4 memory activated, dendritic cells activated, mast cells resting, and neutrophils accounted for a smaller proportion compared to non-tumor cells. On the other hand, T cells follicular helper, T cells gamma delta, macrophages M0, macrophages M1, macrophages M2, and dendritic cells resting were more prevalent in tumors (Fig. 8A). For laryngeal cancer, plasma cells and T cells CD4 memory resting were found in lower proportions among tumor cells compared to non-tumor cells, while T cells CD4 memory activated, macrophages M0, and macrophages M1 showed a higher proportion in tumors (Fig. 8B).

## 4. Discussion

In our study, we identified shared genes between laryngeal cancer and lung cancer, namely UBE2C, POLQ, RAD51, HOXB7, EDNRB, GPD1L, F10, SORBS2, and CXCL12. These genes exhibit high accuracy in diagnosing both types of cancer, providing valuable insights for clinicians in diagnosing, preventing, or treating second primary lung cancer in laryngeal cancer patients. Furthermore, our findings indicate that mutations in these genes increase the susceptibility of laryngeal cancer patients to primary cancer or local recurrence, while also worsening the prognosis of lung cancer patients. This sheds light on why laryngeal cancer patients are more prone to developing second primary lung cancer and underscores the poorer prognosis for patients with both cancers compared to those with pure laryngeal cancer. The relationship between laryngeal cancer and the development of second primary lung cancer is complex, involving multiple transcription factors and miRNAs that primarily act during the cell cycle. Additionally, our study highlights the pivotal role of CXCL12 in tumor microbiota, particularly in inositol phosphate metabolism. The similarity in cell populations between lung cancer and laryngeal cancer, due to their shared respiratory tract, may create a tumor-promoting microenvironment to the development of both cancers. Furthermore, the influence of cell population on metabolism promotes the initiation and progression of laryngeal cancer and lung cancer. Lastly, our findings reveal striking similarities in immune cell infiltration, with macrophages playing a significant role in tumorigenesis and development.

Previous studies have demonstrated that UBE2C regulates cell cycle progression and autophagy through the UBE2C/CDH1/DEPTOR axis in lung cancer, promoting tumor cell progression.^[25]^ Genes such as POLQ and RAD51 play a crucial role in DNA damage repair, which can be both beneficial and detrimental. Deletion or inhibition of these genes can lead to tumor cell formation.^[26–28]^ Additionally, these genes can serve as diagnostic predictors of tumors, aligning with previous literature.^[29,30]^ Tumor formation occurs due to various factors, including mutations in genes (deletions, missenses, etc) during the DNA replication process, coordinated action of multiple transcription factors and miRNAs. Our study found that the mutated group of laryngeal cancer patients had a higher likelihood of developing primary cancer and local recurrence compared to the non-mutated group. Therefore, we speculate that the higher occurrence of second primary lung cancer in laryngeal cancer patients is due to gene mutations shared between the 2 cancers, as they both affect the respiratory tract, which is frequently exposed to airway-related factors. Previous studies have identified smoking, alcohol consumption, and radiation therapy as risk factors for the development of second primary lung cancer in laryngeal cancer patients. Both smoking and radiation therapy can induce gene mutations, supporting our hypothesis.^[12,31]^

Bacterial LPS is a crucial component of the bacterial cell wall and exhibits high bioactivity. LPS functions as a protective barrier for bacteria, enabling them to evade immune recognition through modifications in groups.^[32]^ Previous studies have reported that LPS increases lactate levels in both mouse models and human cells.^[33,34]^ Additionally, LPS-induced changes in histone lysine expression of LINC00152 have been found to promote the occurrence and development of tumors.^[35]^ Another study by Nan Li et al discovered that LPS promotes the expression of CXCR7, leading to the proliferation and metastasis of gastric cancer.^[36]^ CXCR7 serves as the secondary receptor for CXC12. Consequently, we aimed to investigate the role of bacterial lipopolysaccharides in laryngeal and lung cancers. Our study revealed a close association between CXC12 and bacterial lipopolysaccharides, with CXCL12 primarily mediating inositol phosphate metabolism. Notably, inositol phosphate metabolism significantly influences the occurrence and progression of breast cancer.^[37]^ However, the relationship between laryngeal cancer and second primary lung cancer remains unknown.

Single-cell sequencing technology has been widely utilized in various aspects of cancer research, such as determining tumor heterogeneity, identifying cellular properties, discovering novel biomarkers, and investigating molecular and functional strategies.^[38,39]^ In our study, we employed single-cell sequencing technology to identify relevant cell populations in laryngeal and lung cancer tissues. We found that B cells, basophils, CD4+ T cells, CD8+ T cells, dendritic cells, monocytes, neutrophils, NK cells, progenitors, and T cells were active in laryngeal cancer. On the other hand, the main cell populations in lung cancer were intermediate monocytes, classical monocytes, terminal effector CD8 T cells, Th1 cells, non-switched memory B cells, natural killer cells, and BM. Both types of cancer were primarily characterized by immune cell components. Furthermore, we explored the impact of these cell populations on tissue metabolism and discovered that Intermediate monocytes regulate Inositol phosphate metabolism. These monocytes not only play a crucial role in promoting angiogenesis but also contribute significantly to cancer development.^[40,41]^ In the context of cancer, monocyte subsets exhibit various functions that can either promote or fight tumor immunity. These functions include phagocytosis, secretion of tumoricidal mediators, promotion of angiogenesis, remodeling of the extracellular matrix, recruitment of lymphocytes, and differentiation into tumor-associated macrophages and dendritic cells.^[42–45]^ Reham Hammad et al observed that the upregulation of intermediate monocyte frequency and the expression of hsa-miR-21-5p served as sensitive indicators of progression from cirrhosis to hepatocellular carcinoma.^[46]^ Based on our hypothesis, we propose that after the development of laryngeal cancer, the cell population in the lungs becomes relatively active due to the shared respiratory tract. This activation of Intermediate monocytes triggers inositol phosphate metabolism and promotes tumor formation and development.

Previous studies have demonstrated the crucial role of macrophages in the tumor microenvironment, influencing various processes such as angiogenesis, extracellular matrix transformation, cancer cell proliferation, metastasis, immunosuppression, and resistance to chemotherapy and checkpoint blockade immunotherapy.^[47,48]^ Macrophages exist in an undifferentiated state known as M0 and can be polarized into M1 or M2 cell types based on specific signals and the microenvironment. In the initial stages of tumor formation, M1 macrophages within the tumor microenvironment trigger inflammatory and antitumor responses. These M1 macrophages can be induced by cytokines like IFN-γ and TNF-α, or by recognizing bacterial LPS.^[48,49]^ On the other hand, M2 macrophages are induced by Th2 cytokines (IL-4, IL-13, etc.) and exhibit anti-inflammatory and immunomodulatory effects by producing anti-inflammatory factors, thus promoting tumor cell growth.^[50,51]^ In the context of laryngeal cancer patients, it is conceivable that macrophages play a role in anticancer and other immune regulatory processes, while M2 macrophages contribute to cancer development in lung tissue through anti-inflammatory and metabolic pathways.

In addition, the study still has many limits. First, this study employed common bioinformatics analysis methods, lacking any innovative approaches. Second, when conducting tumor biology analysis using different datasets, batch effects may lead to differences in the results. Batch effects may distort the results of gene expression difference analysis, for instance, leading to poor consistency in the identified differentially expressed genes. Third, the sample size of laryngeal cancer dataset and the single-cell datasets were small, and some key clinical factors (age, smoking status, stage, and treatment history) were not provided in the study. It would be verified in larger samples and future studies incorporate clinical data for multivariate analysis. Fourth, the relationship transcription factors, miRNAs, and metabolic pathways and the DEGs were based on bioinformatics predictions, lacking experimental verification. It is suggested that future verification can be conducted through experiments (such as ChIP-seq, qPCR, and metabolomics). In addition, we indeed did not apply our method to validate the composition of single-cell-derived immune cells, which may lead to potential inconsistencies. Future research should consider combining batch RNA sequencing data with single-cell data to more accurately assess the composition of immune cells.

## 5. Conclusion

In conclusion, this study lies in integrating multi-omics data (transcriptome, single-cell, metabolism, and immune infiltration), and for the first time systematically revealed the shared molecular relationship between laryngeal cancer and second primary lung cancer, especially emphasizing the potential role of CXCL12-mediated inositol phosphate metabolism and intermediate monocytes in it. Our findings provide valuable insights for the diagnosis and treatment of laryngeal cancer. However, it is important to acknowledge the limitations of our research, including the lack of experimental and clinical data to validate our hypothesis. Future studies should aim to address this gap and further supplement the topic with sufficient experimental validation.

## Author contributions

**Conceptualization:** Fengfeng Xu, Shiyan Chen.

**Data curation:** Fengfeng Xu.

**Funding acquisition:** Fengfeng Xu.

**Formal analysis:** Tengfei Huang, Qianhui Xu.

**Investigation:** Shiyan Chen.

**Methodology:** Meiqing Zhang.

**Writing – original draft:** Fengfeng Xu.

**Writing – review & editing:** Fengfeng Xu.
